# Spillover Network Features from the Industry Chain View in Multi-Time Scales

**DOI:** 10.3390/e24081108

**Published:** 2022-08-12

**Authors:** Sida Feng, Qingru Sun, Xueyong Liu, Tianran Xu

**Affiliations:** 1 The College of Economics and Management, Beijing University of Chemical Technology, Beijing 100029, China; 2 School of Economics, Hebei University, Baoding 071000, China; 3 School of Management and Engineering, Capital University of Economics and Business, Beijing 100070, China

**Keywords:** volatility spillover, heterogeneous network, time scale, industry chain

## Abstract

Financial stocks in the industry chain interact notably because of close economic and technical relationships. Some participants pay particular attention to one industry chain and are concerned with different investment horizons. The motivation for this study is to offer more targeted information to various market participants who focus on different time scales in one industry chain from a systematic perspective by combining the GARCH-BEKK, heterogeneous network, and wavelet analysis methods. The findings are as follows: (1) For parties who prefer to take more risks to gain higher returns, scale 2 (4–8 days) is a good option, while long-term investment (32–128 days) is suitable for conservative investors. (2) In most cases, some links in the industry chain are particularly sensitive to changes in stocks in other links. (3) The influence, sensitivity, and intermediary of stocks in the industry chain on different time scales were explored, and participants could use the resulting information to monitor the market or select stocks. (4) The structures, key players, and industry chain attributes of the main transmission paths differ on multi-time scales. Risk transmission can be controlled by intercepting important spillover relations within the paths.

## 1. Introduction

Interactions among financial stocks, which are caused by risk transmission and information transfer, are known as the volatility spillover effect [1,2,3]. This phenomenon is reflected in the volatility relationship within financial time series [4]. The volatility spillover effect is a widely discussed topic, and many studies on it have been conducted [5,6,7]. However, there are various stocks in the market, and the volatility of one might cause volatility within the whole [8]. For example, if stock A is volatile, it will directly affect other stocks. The affected stocks, in turn, influence other stocks. Volatility transfer may occur several times. As a result, the whole system might be influenced by the volatility of stock A. Therefore, a systematic view could help market participants to comprehensively understand the stock market. In recent years, scholars began to construct financial networks and study network features to explore the spillover effects among stocks from a systematic perspective [6,7,9].

Stocks in the same industry can logically form a chain based on their economic and technical relationships, i.e., the industry chain [10]. Studying the stocks from an industry chain perspective is necessary because it can provide more targeted information to market participants about a particular industry. There are various types of stocks in the market which can be classified into industries; some participants pay more attention to a particular industry. Due to value exchange, the stocks of companies in the same industry chain are closely linked [11]. Links in the industry chain can be divided into three types: upstream, midstream, and downstream. Accordingly, a company might be affected by one or more types of link. For instance, some stocks belong to upstream, and some belong to midstream, and others belong to downstream. If the network nodes have different features, the network is regarded as heterogeneous. Therefore, from an industry chain perspective, the spillover network of stocks in the industry is also heterogeneous.

However, only a few studies have provided stock network analyses from an industry chain perspective. These studies have successfully used heterogeneous networks to explore the chain features. For instance, Zhang [10] constructed an influence index threshold network model using the Pearson correlation coefficient and industrial chain information about China’s PV market to identify leading enterprises. Jia [12] tested the daily closing price time series of stocks in the global rare earth industry chain using the Granger causality test and constructed a risk transmission network of the industrial chain to explore essential stocks and transmission paths. Feng [11] studied risk transmissions among different links in the electric vehicle industrial chain using GARCH-BEKK, motif analysis and network analysis. By dividing the sectors into different links in the industry chain, Xu [13] investigated the interdependence of tail risks among industries in the Chinese stock market by constructing a tail event-driven network. 

Different market participants, such as individual investors, fund managers, and policymakers in one industry chain, are concerned with varying investment horizons [14]. For instance, investors are mainly interested in short investment horizons, while policymakers pay more attention to long-term equilibrium [15]. The financial time series comprises different frequency components, forming a multiscale conformation relative to a raw time series [16]. The stocks in different time horizons have their own distinct features [17], as do the corresponding spillover networks of stocks. Therefore, decomposing the time series of one industry into different time horizons can reveal useful hidden information and offer specific information to participants who are focused on distinct time horizons in the industry chain. However, previous network analyses of the stock market from an industry chain perspective have ignored the differences based on different time scales. 

Wavelet analysis can transform an original financial time series into different time scales. This method has been successfully used to reveal hidden information in time series [18,19,20]. For instance, using wavelet analysis, Pascoal [19] studied market efficiency, roughness, and long memory in the PSI20 Index. Wang [16] detected the correlation characteristics between financial time series based on multi-resolution analysis by decomposing a raw time series into eight scales. Fernandez [21] investigated the spillover relations in multi-time scales using wavelet analysis and GARCH. Liu [22] used maximum discrete overlapping wavelet transform to decompose Wind Global Market Indices sectors into six time scales and estimated their spillover relations on different time scales using GARCH-BEKK. Therefore, aiming to reveal the hidden information of spillover relations among stocks in the industry chain, and by providing distinct market participants with more specific references on different time horizons, this study uses wavelet analysis to decompose an original time series into several multi-scale time series.

As for the spillover effect measurement, GARCH-BEKK, proposed by Engle and Kroner [23], is applied in this study. Engle is a Nobel Prize winner in Economics because of his contribution to Autoregressive Conditional Heteroskedasticity (ARCH). ARCH can measure the variation of time series variables [24]. GARCH-BEKK is one of the models in the ARCH model family; the advantage of this model is that there is no restriction on the correlation structure between the variables [25]. GARCH-BEKK is widely used to study volatility spillover in financial markets [5,26,27,28].

Herein, the lithium-ion battery industry chain in China is taken as a case study. Lithium-ion batteries are regarded as the most promising technology for developing power sources for electric vehicles [29]. In recent years, many countries, including China, have been proactively developing their lithium-ion battery industries [30]. Therefore, this industry has gained a great deal of attention from stock market participants. 

In summary, the motivation of this study is to offer more pertinent information to distinct market participants who are focused on different time scales in a given industry from a systematic perspective. This research takes the lithium-ion battery industry chain in China as a case study and explores the multi-scale spillover relations in the industry chain by combining spillover measurement, heterogeneous network, and wavelet analysis. First, the raw time series of stocks in the lithium-ion battery industry chain are decomposed into multi-time scales by wavelet analysis to extract the hidden frequency information in the original series. Second, the spillover effects of stocks in the industry chain on different scales are measured by GARCH-BEKK. Third, heterogeneous spillover networks are constructed according to the links (upstream, midstream, and downstream) to which the stocks belong. The stocks are nodes, and the spillover relations are edges. Fourth, the topological features, including structural entropy, key stocks, and main transmission paths in distinct scales, are studied to answer four questions: (1) Which time scale has the highest or lowest spillover strength? (2) How do the stocks in one link affect other links in the industry chain on different scales? (3) What are the typical stocks in the industry chain regarding influence, sensitivity, and intermediaries on distinct scales? (4) What are the main transmission paths on the six applied scales?

## 2. Data and Methodology

### 2.1. Data

This paper collected the daily closing price data for 59 listed companies in the lithium-ion battery industry chain from the Wind database (download date: 11 May 2022). Two companies with large amounts of missing data were removed. Then, according to industry research reports, 57 companies were classified as follows: 12 upstream companies, 25 midstream companies, 15 downstream companies, and 5 companies that belong to more than one link in the industry chain (see Appendix A). The data time range was from 17 September 2020 to 10 May 2022. 

### 2.2. Methodology 

First, the logarithmic return of each company’s stock was calculated to ensure the stationary nature of the data. Second, wavelet analysis was used to decompose the original time series into distinct time scales to analyze the relationships of stocks in the lithium-ion battery industry chain from a multi-scale perspective. Third, by using the GARCH-BEKK model, the spillover relationships between each pair of stocks in different time scales were calculated. Fourth, spillover networks in the industry chain on different scales were constructed, and the characteristics of spillover relationships under different time scales were explored using network indexes.

Formula (1) calculates the logarithmic return series of each company’s stock in the industry chain.

(1) $$R_{i}=\ln\left(P_{i,t}\right)-\ln\left(P_{i,t-1}\right)$$

where $R_{i}$ is the logarithmic return of company I, $P_{i,t}$ is the daily closing price of company i on day t, and $P_{i,t-1}$ is the daily closing price of company i on day t − 1. 

#### 2.2.1. Time Scale Decomposition by Maximal Overlap Discrete Wavelet Transformation (MODWT)

To reveal the spillover relationships among stocks in different frequencies and offer pertinent suggestions to various market participants focusing on different time horizons, this study used the Maximal Overlap Discrete Wavelet Transformation wavelet (MODWT) (For more details of the instruction to MODWT, see Percival and Walden (2000) [20]) method to decompose the original logarithmic return series into different time scales. This process gave us the following advantages: (1) MODWT overcomes the unfavorable effects caused by starting point selection for analyses; and (2) The data were not required to have a dyadic length [31,32,33]. The original logarithmic return series X of each stock in the industry chain could be decomposed and reconstructed into several scales and one trend level.

(2) $$X=\sum_{j=1}^{J}D_{j}+S_{J}$$

where D_j_ is the wavelet details at scale j and $S_{J}$ is the trend level. Referring to previous research using wavelet analysis to study daily financial data, J was set at 6 [22,34,35]. The original time series were decomposed into six subsequences (Scales 1, 2, 3, 4, 5, and 6) by MODWT. Each subsequence represents the contribution of fluctuations on a specific scale. When working with daily data, scale 1 means 2–4 day dynamics; Scale 2 indicates 4–8-day dynamics.; Scale 3 represents 8–16 day dynamics; Scale 4 indicates 16–32 day dynamics; Scale 5 means 32–64 day dynamics; and Scale 6 corresponds to 64–128 day dynamics [21]. Scales 1 and 2 can be regarded as short-term, Scales 3 and 4 are medium-term, and Scales 5 and 6 are long-term (Table 1).

#### 2.2.2. Spillover Relationship Estimation Using the GARCH-BEKK Model

GARCH-BEKK [23] is widely used to study volatility spillover in financial markets [5,26,27,28]. One of the strengths of this model is that there are no restrictions on the correlation structure between the variables [25]. This study used GARCH-BEKK to measure the spillover relationship between stocks and the chosen one-time lag. The GARCH-BEKK model applied in this study is as follows:

Mean equation:

(3) $$R_{t}\left(j\right)=\left[\begin{array}{c} R_{1,t}\left(j\right)\\ R_{2,t}\left(j\right) \end{array}\right]=\left[\begin{array}{c} \mu_{1}\left(j\right)\\ \mu_{2}\left(j\right) \end{array}\right]+\left[\begin{array}{cc} \varphi_{11} & \varphi_{12}\\ \varphi_{21} & \varphi_{22} \end{array}\right]\left[\begin{array}{c} R_{1,t-1}\left(j\right)\\ R_{2,t-1}\left(j\right) \end{array}\right]+\left[\begin{array}{c} \epsilon_{1,t}\left(j\right)\\ \epsilon_{2,t}\left(j\right) \end{array}\right]$$

where $R_{t}\left(j\right)$ is a $\left(2\times 1\right)$ vector of the logarithmic return of Stocks 1 and 2 at time t in scale j, $\epsilon_{1,t}\left(j\right)$ and $\epsilon_{2,t}\left(j\right)$ are the random errors at time t in scale j, and $\epsilon \left(t\right){|}_{\Omega_{t-1}}=\left[\epsilon_{1,t}\left(j\right),\epsilon_{2,t}\left(j\right)\right]\prime ∼N\left\{0,H_{t}\left(j\right)\right\}$, and $\mu_{1}\left(t\right)$ and $\mu_{2}\left(t\right)$ are the long-term drift coefficients of Stocks 1 and 2 in scale j.

Variance equation:

(4) $$H_{t}\left(j\right)=C\prime \;C+A\prime \epsilon_{t-1}\left(j\right)\epsilon_{t-1}^{\prime }\left(j\right)A+{B\prime H}_{t-1}\left(j\right)B$$

(5) $$C=\left[\begin{array}{cc} c_{11} & 0\\ c_{21} & c_{22} \end{array}\right],\;A=\left[\begin{array}{cc} a_{11} & a_{12}\\ a_{21} & a_{22} \end{array}\right]\;\mathrm{and}\;B=\left[\begin{array}{cc} b_{11} & b_{12}\\ b_{21} & b_{22} \end{array}\right]$$

where C is a (2 × 2) constant matrix for lower triangular, $H_{t}\left(j\right)$ is the conditional variance-covariance matric at scale j, A and B are the coefficients of, respectively, the conditional residual matrix and the conditional covariance matrix. Diagonals $a_{11}$, $a_{22}$, $b_{11}$, and $b_{22}$ represent the effects of own previous shocks and volatility. Off-diagonals $a_{12}$, $a_{21}$, $b_{12}$, and $b_{21}$ mean shocks and volatility between two stocks.

The BHHH algorithm of the maximum likelihood estimation method was used to estimate the model. The conditional log likelihood function is as follows:

(6) $$L\left(\theta \right)=-\mathrm{Tln}\left(2\pi \right)-\frac{1}{2}\sum_{t=1}^{T}\left[\ln\left|H_{t}\left(\theta \right)\right|+\epsilon_{t}{\left(\theta \right)}^{\prime }H_{t}^{-1}\epsilon_{t}\left(\theta \right)\right]$$

where T is the number of observations and θ is the vector of the parameters to be estimated. 

As mentioned above, $a_{12}$ and $b_{12}$ are the shocks and volatility, respectively, between two stocks. $a_{12}$ is the shock effect (ARCH effect) from stock 1 to stock 2, and $b_{12}$ is the volatility transmission (GARCH effect) from stock 1 to stock 2 [23,36]. The shock effect and volatility transmission can be demonstrated by the absolute values of $a_{12}$ and $b_{12}$. This study explores the total spillover effect between stocks; therefore, the total volatility spillover effect from stock 1 to stock 2 can be calculated by summing $\left|a_{12}\right|$ and $\left|b_{12}\right|$, as discussed in previous studies [27,37,38]. The formula is as follows:

(7) $${\mathrm{Spillover}}_{12}=\left|a_{12}\right|+\left|b_{12}\right|$$

#### 2.2.3. Heterogeneous Spillover Network of an Industry Chain 

**Figure 1:**
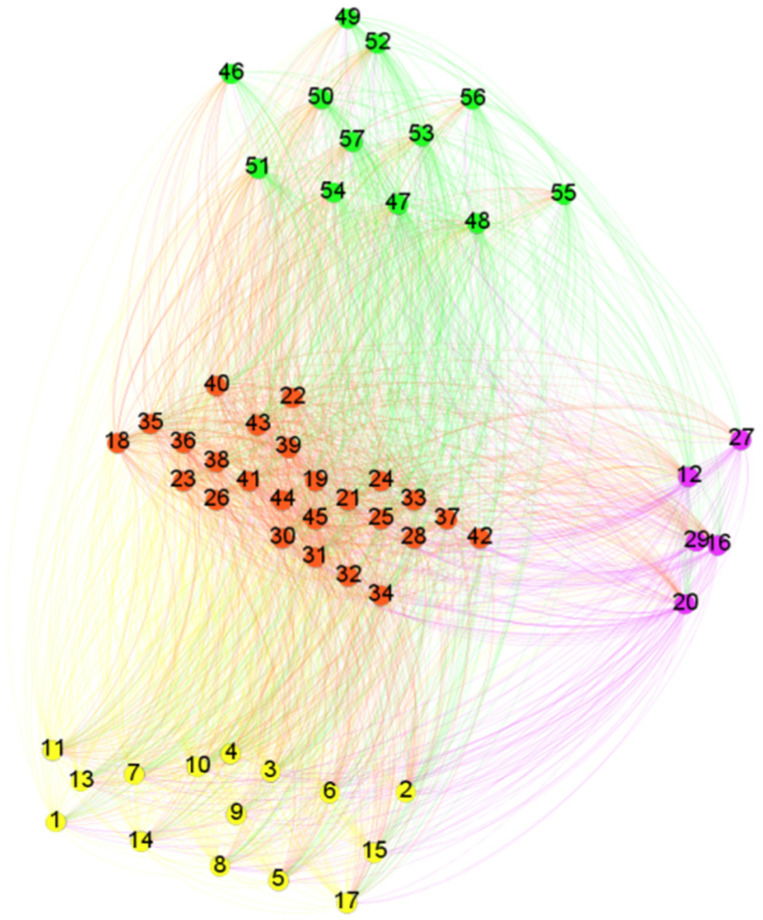
The spillover network of stocks in the lithium-ion battery industry in Scale 1. The nodes are stocks in the lithium-ion battery industry chain. The edges are spillover relations. The weight of an edge is the spillover strength, as obtained in Section 2.2.2, and the direction of an edge indicates the spillover direction. The colors represent the industry chain attribute of the stock. Green represents upstream, red means midstream, and yellow is downstream. The stocks in purple are classified into more than one link.

An industry chain network is revealed by constructing a heterogeneous network. In this case, the nodes are stocks in the lithium-ion battery industry chain. The edges are spillover relations. The weight of an edge is the spillover strength, as obtained in Section 2.2.2, and the direction of an edge indicates the spillover direction. The color represents the industry chain attribute of the stock. Green represents upstream, ted means midstream, and yellow is downstream. The stocks in purple are classified into more than one link. Six spillover networks are constructed. Figure 1 shows the spillover network of the lithium-ion battery industry chain in Scale 1. The network indexes were applied to analyze the spillover features.

(1) Stock sensitivity 

The sensitivity of a stock measures how sensitive the stock is to fluctuations of other stocks in the industry chain. It can be calculated by the total spillover strength it receives from others.

(8) $$S\left(j\right)=\sum_{i=1,i\neq j}^{N-1}w_{\mathrm{ij}}$$

where $S\left(j\right)$ is the sensitivity strength of stock j and $w_{\mathrm{ij}}$ is the quantity of spillover from i to j.

(2) Stock influence

The influence of a stock is the total spillover strength it sends to others, which can be defined as follows:

(9) $$I\left(j\right)=\sum_{i=1,i\neq j}^{N-1}w_{\mathrm{ji}}$$

where $I\left(j\right)$ is the influence strength of stock j and $w_{\mathrm{ji}}$ is the quantity of spillover from j to i.

(3) Stock intermediary

The intermediary ability of a stock can be measured by betweenness centrality. The formula is as follows:

(10) $$B\left(i\right)=\sum_{j}^{n}\sum_{k}^{n}T_{\mathrm{jk}}\left(i\right),j\neq k\neq i,j<k,T_{\mathrm{jk}}\left(i\right)=P_{\mathrm{jk}}\left(i\right)/P_{\mathrm{jk}}$$

where B(i) is the betweenness centrality of stock I, $T_{\mathrm{jk}}\left(i\right)$ is the probability that stock i is on the shortest path from stock j to stock k, $P_{\mathrm{jk}}\left(i\right)$ is the shortest path with stock i and $P_{\mathrm{jk}}$ is the number of shortest paths, m is the number of stocks, and n is the number of shortcut paths between stock j to stock k.

(4) Structure entropy of influence, sensitivity, and intermediary

Structure entropy is an index combing network analysis and entropy theory. It is widely applied to measure heterogeneity within a network index [7,39,40]. The influence structure entropy, sensitivity structure entropy, and intermediary structure entropy are calculated to respectively depict differences of influence, sensitivity, and intermediary among stocks in a network on different time scales [41]. The formulas are as follows:

(11) Influence structure entropy=−∑j=1nIjIlnIjI

(12) Sensitivity structure entropy=−∑j=1nSjSlnSjS

(13) Intermediary structure entropy=−∑j=1nBjBlnBjB

where I is the sum of influence of all the stocks, S is the sum of sensitivity of all the stocks, and B is the the total intermediary of all the stocks. 

(5) Network influence range and strength

The network influence range is the average of the stocks’ influence ranges, which is measured by the out degree.

(14) $$\mathrm{Netinrange}=\frac{1}{N}\sum_{m=1}^{N}\sum_{i=1,i\neq j}^{N-1}e_{\mathrm{ji}}$$

where N is the number of stocks in the network and $e_{\mathrm{ti}}$ is the relationship between j and i. If there is a spillover relation from j to i, $e_{\mathrm{ji}}=1$; if not, $e_{\mathrm{ji}}=0$.

The network influence strength is the average of the stocks’ influence index, as calculated using Formula (9). The formula is:

(15) $$\mathrm{Netinstr}=\frac{1}{N}\sum_{j=1}^{N}I\left(j\right)$$

where N is the number of stocks in the network and $I\left(j\right)$ is the influence of stock j.

(6) Maximum spanning tree

The maximum spanning tree is applied to extract the main paths of spillover transmission. It comprises a tree of all nodes with a maximum weight sum [42]. A similar concept, named minimum spanning tree, comprises a tree of all nodes with a minimum weight sum. In some financial networks constructed by distance, short distance (small weight) means high similarity, so a minimum spanning tree is applied [43,44], while in other cases, including this study, larger weights are prioritized. In this study, we sought to extract the paths which convey the most information. As such, we focused on the edges with larger weights. Researchers typically extract maximum spanning trees according to edges with larger weights [45,46,47]. As for the extraction process, the difference between the aforementioned kinds of trees is that the minimum spanning tree chooses the edges according to ascending order of weight, while the maximum spanning tree does so according to descending order. Many algorithms can be used to calculate the minimum spanning tree, and all of them can be easily transformed to calculate the maximum spanning tree [48]. This study used the Kruskal algorithm to extract the maximum spanning tree as follows [48]: First, the edges of the spillover network were sorted into decreasing order according to their spillover strength. We then added the first edge to the maximum spanning tree. Next, we added the next edge to the tree if and only if it did not form a cycle in the current tree without considering the direction of the edges. If the tree had N−1 edges, the process ended and the maximum spanning tree was obtained. Otherwise, the previous step was repeated.

## 3. Empirical Results and Discussion

### 3.1. Overall Spillover Network Features on Distinct Time Scales

**Figure 2:**
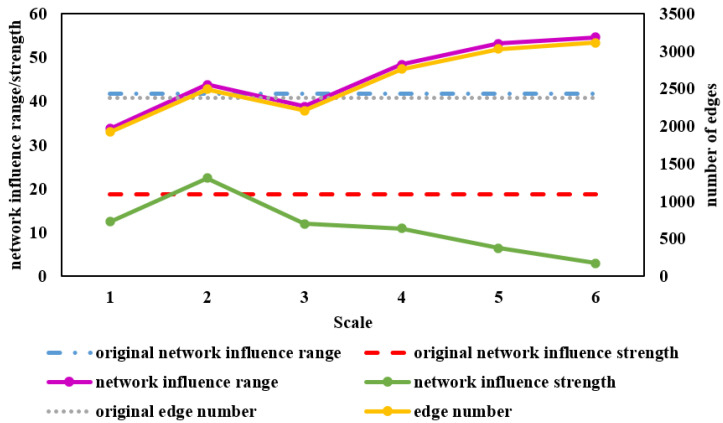
Spillover network features on distinct time scales. The abscissa represents each scale. The left ordinate represents the network influence range or strength, and the right ordinate indicates the number of edges.

Network features, namely, the number of edges, network influence range, and network influence strength of different time scales, are shown in Figure 2. The indexes of the original time series are also displayed as benchmarks, shown as broken lines. The abscissa represents each scale. The left ordinate represents the network influence range or strength, and the right ordinate indicates the number of edges. 

The overall trend of the number of edges was consistent with the network influence range, which first increased at Scale 2, then decreased at Scale 3, and finally continued to increase, exceeding the original level. As for the network influence strength, it was consistent with the trend of the influence range and edge number from Scale 1 to Scale 3, but it decreased continuously from Scale 4 to Scale 6 and remained lower than the original network. Although the network influence ranges from Scale 4 to Scale 6 were expanding and became higher than the original level, the network influence strength index was decreasing and eventually fell below the original level. This implies that in the medium-term and long-term, the range of risk and volatility transmission among stocks was expanding while the intensity became weaker over time.

The range and strength of Scale 2 were more extensive than in the original network. In addition, this was the scale with the highest network influence strength and the only scale that exceeded the influence strength of the original network. This indicated that the intensity of the risk and volatility transmission of Scale 2 was the strongest. High risk means potentially higher returns [4], so Scale 2 is suitable for those who are willing to take more risks to gain higher returns. In contrast, Scales 5 and 6, which are long-term, showed the lowest network intensity. This corresponds with the widely accepted notion that long-term investments are more stable [14]. Therefore, Scales 5 and 6 are more suitable for risk avoiders.

### 3.2. Influence between Two Links on Distinct Time Scales

**Figure 3:**
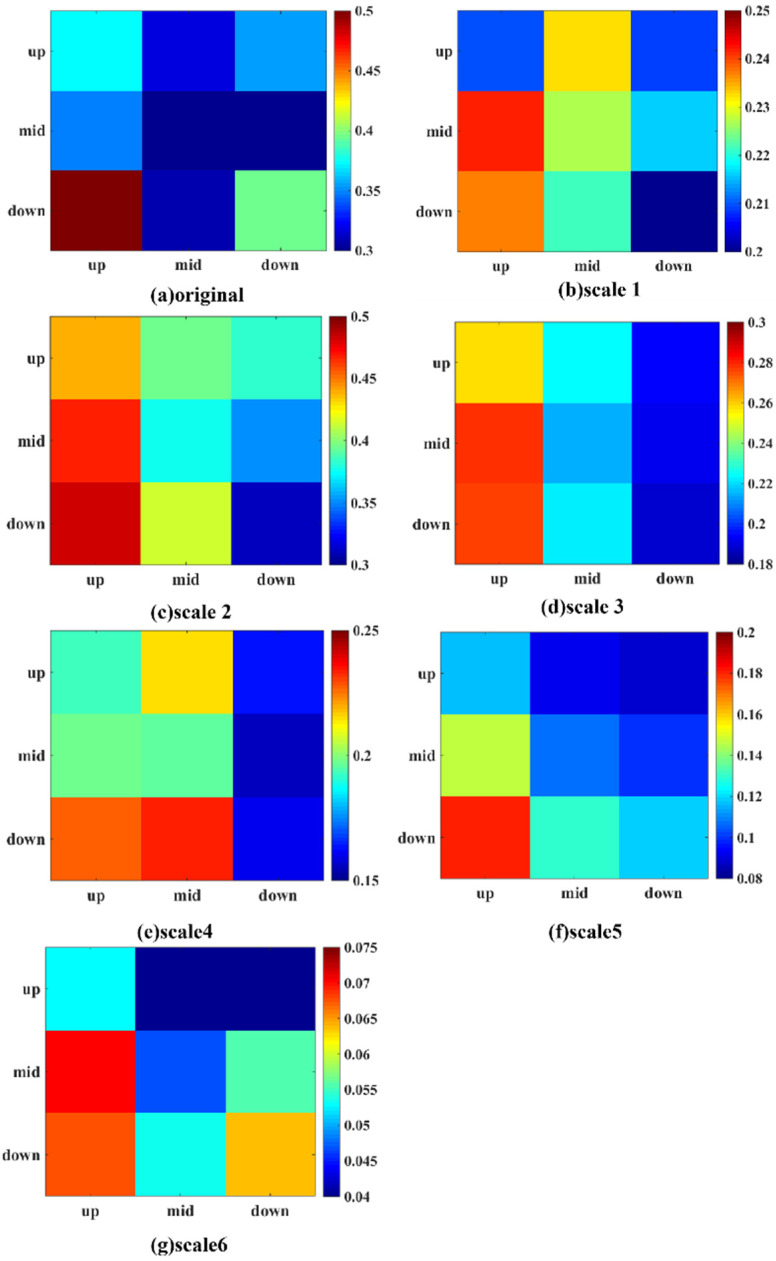
Influence between two links in different time scales. The ordinate and the abscissa indicate the links of upstream, midstream, and downstream. The color of each block in the figure represents the relative spillover strength of the stocks from the link in the ordinate to the link in the abscissa; the lower the value, the colder the color.

The spillover strength between two links of the industry chain on different time scales is shown in Figure 3. The ordinate and the abscissa indicate the links of upstream, midstream, and downstream. Stocks belonging to more than one link were classified to the corresponding links, so the number of stocks in upstream, midstream, and downstream were 16, 27, and 18. The numbers of stocks in distinct links were different, so the relative spillover strength, i.e., the average spillover strength of the stocks in one link relative to another, was used to compare the influence of one link upon another. This was obtained by the total spillover strength from link A to link B divided by the number of stocks in link A. The color of each block in the figure represents the relative spillover strength of the stocks from the link in the ordinate to the link in the abscissa; the lower the value, the colder the color. For example, for the left bottom block in Figure 3a, the abscissa is upstream and the ordinate is downstream, so this block indicates the relative spillover strength from the downstream to the upstream. The color of this block is the hottest in Figure 3a, indicating that the average spillover influence of stocks from the downstream to the upstream was the highest in the original data. 

The average spillover influence of stocks in different links on each time scale can be found by referring to each column. Participants who focus on a certain link can pay attention to the average influence of stocks in different links. For instance, in Time scale 2 (Figure 3c) for upstream, the average spillover influence from the downstream stocks was the largest. The average spillover influence from the downstream stocks was the strongest for the midstream. As for downstream, the average spillover influence from the upstream stocks was the largest. Therefore, upstream and midstream market participants should pay special attention to changes in downstream stocks, while downstream participants should focus on changes in upstream stocks. 

Overall, we can founds that the spillovers between links on distinct time scales show different features. Therefore, participants concerned with varying horizons could obtain more targeted information by referring to the corresponding time scale, indicating the necessity of multiscale studies. The blocks on the diagonal line from the bottom left to the top right in each time scale are not the hottest in each column, except the three blocks from downstream to downstream in the original time series, Scales 5 and 6. This means that except for the downstream in the original time series, Scales 5 and 6, the most extensive average influence was from the stocks in another link rather than those in Link A. This showed that in most cases, on average, a particular link is more sensitive to changes of stocks in other links in the lithium-ion battery industry. This is consistent with our previous research investigating the spillover relationships in the electric vehicle industry chain without considering multiple time scales [11]. Therefore, attention should be paid to other links; this reflects the importance of a whole-industrial-chain perspective.

### 3.3. Influence, Sensitivity, and Intermediary of Stocks on Distinct Time Scales

**Figure 4:**
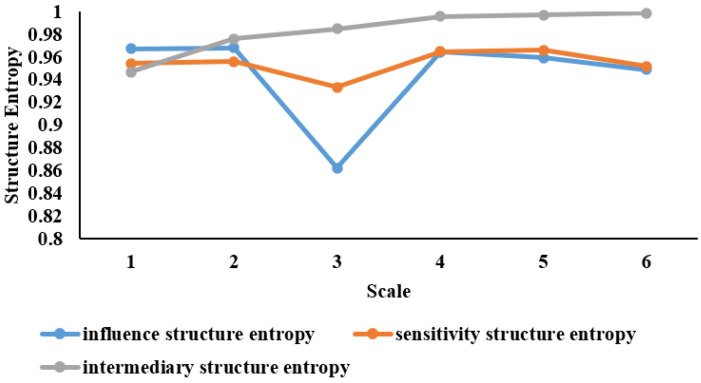
The structure entropy of sensitivity, influence and intermediary. The abscissa represents each time scale and the ordinate represents the structure entropy value.

This section explores three attributes: sensitivity, influence, and intermediary of stocks in the industry chain on six time scales. The structure entropy of sensitivity, influence, and intermediary from Scale 1 to 6 are displayed in Figure 4. The abscissa represents each time scale, and the ordinate represents the structure entropy value. 

As shown in Figure 4, the intermediary structure entropy increases from Scale 1 to Scale 6, indicating that the intermediary becomes more and more evenly distributed over time. The influences structure entropy and sensitivity structure entropy showed similar trends, with a sharp drop in Scale 3, especially for influence structure entropy. This means that the distribution of influence and sensitivity in Scale 3 is the most inhomogeneous among all time scales. The stocks showed relatively noticeable differences in terms of influence and sensitivity, and as such, would be a rather good choice for investment portfolios seeking to hedge against risks.

**Figure 5:**
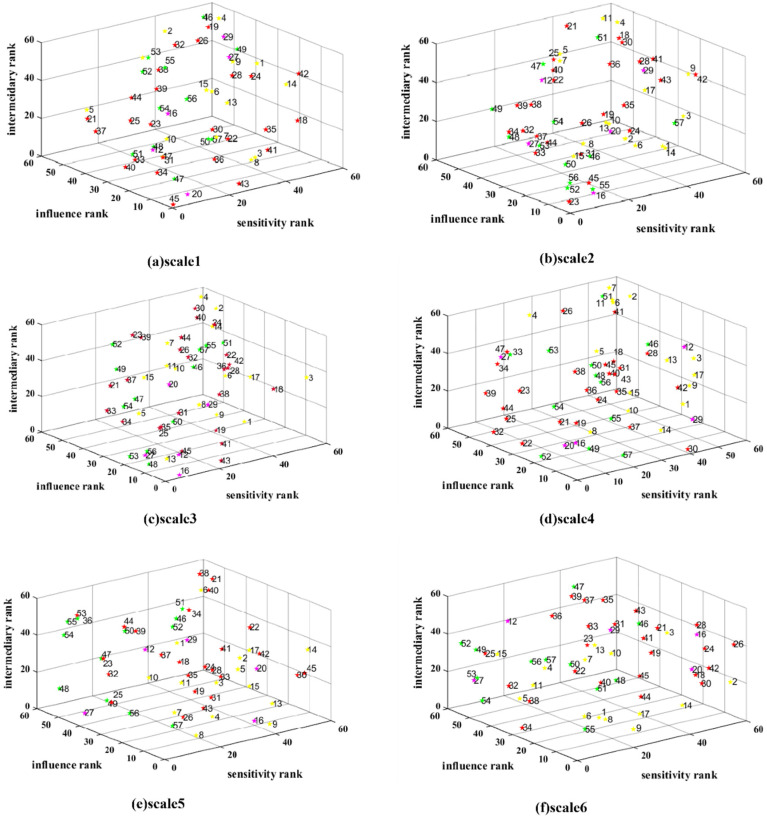
Influence, sensitivity, and intermediary ranks of stocks. Dots in the plot represent stocks. The X axis is the ranking of sensitivity among the stocks in descending order. The Y axis is the influence ranking, and the Z axis is the intermediary ranking. The color of the stock represents the industrial chain link attribute. Green represents upstream, red indicates midstream, and yellow means downstream. Purple stocks belong to more than one link. The number near the stock is the stock ID. The names of the stocks can be found in the Appendix A.

In detail, Figure 5 shows six three-dimensional scatter plots from Time scales 1 to 6. The dots in the plot are stocks. The X axis is the ranking of sensitivity among the stocks in descending order. The Y axis is the influence ranking, and the Z axis is the intermediary ranking. The color of the stock represents the industrial chain link attribute. Green represents upstream, red indicates midstream, and yellow means downstream. Purple stocks are belong to more than one link. The number near the stock is the stock ID. The name of each stock can be found in the Appendix A.

Overall, the distributions of stock features are different from Scale 1 to Scale 6. From an industry chain perspective, as shown in Table 2, it is interesting that stocks belonging to more than one link have the strongest influence, on average, from Scale 1 to Scale 5. This implies that expanding business across the links, known as vertical integration, could enhance the degree of influence within the industry chain.

There were stocks with high rankings in all three aspects in Scales 1, 2, and 3. No such stock existed on Scales 4 to 6. This means that in Scales 1, 2, and 3, a small number of stocks play essential roles in all three aspects. In Scale 1, the stock near (0,0,0) was 45 CHUANGXIN in midstream, ranking first in terms of influence, sensitivity, and intermediary. The rankings of 45 CHUANGXIN were also relatively high in Scale 2, ranking first in influence, 7th in sensitivity, and 13th in intermediary. Meanwhile, 45 CHUANGXIN is a leading supplier of lithium-ion battery diaphragms. In the first half of 2021, the company shipped about 1.2 billion square meters of wet lithium battery diaphragm, making it the world’s largest supplier and giving it the world’s largest market share [49]. In Scale 2, 23 SHANDONG SHIDA SHENGHUA CHEMICAL GROUP appeared around the (0,0,0) corner, with rankings of 5, 7, and1, respectively (midstream). This company is a leading producer of electrolytes for lithium-ion batteries [50]. Its predecessor was a school-run enterprise at the China University of Petroleum (East China), a national “double first-class” discipline construction university. Globally, it is the only company that can simultaneously provide electrolyte solvent, solute, and additive products for lithium-ion batteries [51]. In Scale 3, the top stock was 16 DFD (6,1,1), which was both midstream and downstream. DFD has independently developed preparation technique for high-purity crystal lithium hexafluorophosphate. The purity, quality, stability, and other indicators of the product are better than those produced elsewhere domestically, making it essential in the lithium-ion battery chain [52]. Recently, the company also accelerated the layout of the downstream lithium-ion battery field. 

Table 3 demonstrates the typical stocks in terms of influence, sensitivity, and intermediary. In Scale 1, 45 CHUANGXIN and 47 SINOMINE were shown to be highly influential and sensitive. As mentioned above, 5 CHUANGXIN had the highest intermediary; therefore, market participants should pay close attention to this stock. As shown in Section 3.1, Scale 2 is suitable for risk seekers because of its high spillover strength. In Scale 2, 33 DYNANONIC, 48 WEIHUA, and 49 YONGXING MATERIAL were shown to have the highest sensitivities. DYNANONIC produces nanometer lithium iron phosphate, which is a type of anode material [53], while 48 WEIHUA and 49 YONGXING MATERIAL supply Li-ion battery materials [54,55]. In general, higher risk means higher returns, so risk seekers in Scale 2 could choose these stocks.

As shown in Section 3.1, the risk is lower in Scales 5 and 6. The stocks with the lowest sensitivity are shown in Table 2. Interestingly, in Scale 5, 45 CHUANGXIN is the antepenultimate stock in terms of sensitivity. This contrasts sharply with the top-ranking enterprise in Scales 1 and 2. Moreover, 2 BAOLI NEW, the most influential player in Scale 6, has influence rankings of 57th, 34th, 47th, 50th, and 9th from Scales 1 to 5. It was also the antepenultimate stock in terms of sensitivity in Scale 6. This indicates that 2 BAOLI NEW, which has a low level of influence on other scales, has the most influence in Scale 6 and is not easily influenced by other stocks due to its low sensitivity. The main products of BAOLI NEW are battery management systems (BMS), power batteries, and energy storage battery packs [56].

As for the intermediary, we found that although some stocks do not have strong influences, they also play important roles as intermediaries. In situations of significant change, media stocks can be controlled to reduce fluctuations. For instance, in Scale 2, 55 CTM and 46 ZANGGE MINING ranked 11th and 33rd, respectively, in terms of influence, while they were still essential because they play mediating roles. CTM primarily produces copper, cobalt, and nickel, i.e., critical raw materials for the downstream new energy vehicle industry [57]. ZANGGE MINING also supplies battery-grade lithium carbonate, placing it upstream in the lithium-ion battery chain.

### 3.4. Main Transmission Paths on Different Time Scales 

**Figure 6:**
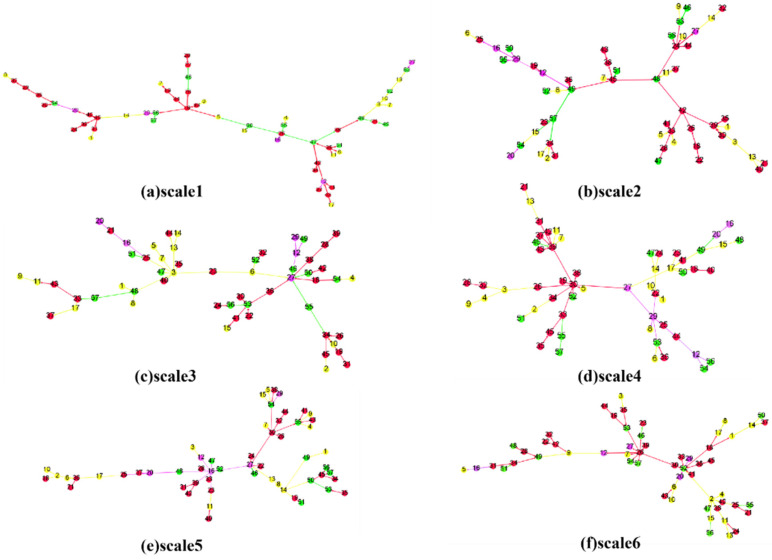
The main transmission paths on different time scales. The colors of the nodes represent the stock industry chain attributes. Green means the stock belongs to the upstream of the industry, red indicates the midstream, yellow represents downstream, and purple indicates that the stock belongs to more than one link in the industry chain. The edge colors indicate the source stock’s corresponding industry chain attribute in the spillover relation.

The main transmission paths on different time scales were extracted using a maximum spanning tree, shown in Figure 6. The colors of the nodes represent the industry chain attributes of the stock. Green means the stock belongs to the upstream, red indicates midstream, yellow represents downstream, and purple nodes indicated that the stock belongs to more than one link in the industry chain. The edge color indicates the source stock’s corresponding industry chain attribute in the spillover relationship.

Overall, the main transmission paths on different scales show different characteristics. There were two main clusters in Scale 4, while in Scale 1, chain distribution was observed. The stocks on the central paths contain different industry chain features on different scales. In Scale 3, the downstream stocks are in the central position, while in other cases, they tend to be scattered around the margins. In Scale 5, the stocks on the central path belonging to more than one link in the industry chain. This implies that the structures, key players, and industry chain attributes in the main transmission paths have significant differences over different time scales. 

The maximum or minimum spanning tree is most commonly used to simplify complex financial networks [1]. The entire network will be more effectively controlled if these critical paths are controlled [4], i.e., the spillover relations on the main path of the tree can be controlled to cut off risk transmission. Taking Scale 2 as an example, if 45 CHUANGXIN undergoes severe fluctuation, the relationships among 45 CHUANGXIN and 48 WEIHUA or 49 YONGXING MATERIALS can be cut to control the risk transmission. 

## 4. Conclusions

Aiming to offer more targeted information to market participants who wish to focus on distinct time scales or links in the industry chain, this study combined the method of GARCH-BEKK, heterogeneous network, and wavelet analysis to reveal the distinguishing spillover features of stocks on different time scales from an industry chain perspective. The findings can be summarized as follows:

(1) Investors could choose investment time horizons according to the risk transmission on distinct scales. For investors preferring to take more risks to gain higher returns, scale 2 (4–8 days) is a good option. The risk transmission among stocks in time scale 2 (4–8 days) has the highest spillover strength, and therefore, it is the most active and risky. In contrast, for conservative investors, the long term (32–64 days and 64–128 days) is preferred. The spillover relations in Scales 5 and 6 are the most significant, but the spillover strengths are the lowest. This means that in the long term (32–64 days and 64–128 days), the connections of stocks are greater, while the intensities of interactions are the lowest. Therefore, investors could use this information to choose a suitable investment time horizon. 

(2) Participants who focus on a particular link should also be aware of the impact of the stock price changes within this link. As for downstream, in the long term, stock in the same link has, on average, the greatest effect. Related participants who focus on the downstream in the long term should keep a close eye on downstream stock price changes. In most cases, a certain link is more sensitive to stock changes in other links. Except for the three blocks from downstream to downstream in the original time series in Scales 5 and 6, the blocks on the diagonal line from the bottom left to the top right in each time scale are not the hottest. Therefore, attention should also be paid to other links in the industry. 

(3) The influence, sensitivity, and intermediary of stocks in the industry chain on six time scales were explored. Overall, Scale 3 (8–16 days) was found to be a relatively good choice for investment portfolios seeking to hedge against risk, because it has the lowest influence structure and sensitivity structure entropies. From Scales 1 to 5, on average, stock belonging to more than one link has the highest influence value. This implies that vertical integration could increase the degree of influence in the industry chain. Some stocks play essential roles in all three aspects, i.e., influence, sensitivity, and intermediary, from Scales 1 to 3; these are leading stocks in their field. The volatility of stocks with strong influence should be closely monitored. As for intermediary, in case of significant changes, media stocks with high intermediary ability can be assessed to reduce fluctuations. Investors can use sensitivity to select stocks. Risk seekers could choose stocks with high sensitivity, such as 33 DYNANONIC, 48 WEIHUA, or 49 YONGXING MATERIAL in Scale 2. Risk avoiders should pick stocks with lower sensitivity, such as 2 BAOLI NEW, 42 GREAT SOUTHEAST, or 26 FULIN PM in Scale 6.

(4) Risk transmission can be controlled by identifying the important spillover relationships within the main paths, such as those from 45 CHUANGXIN to 48 WEIHUA and 49 YONGXING MATERIALS. The structures, key players, and industry chain attributes on the main transmission paths show considerable differences on different time scales. Market participants should keep a close eye on the corresponding time scale. For instance, the yellow stocks are in the margin positions in most cases except for Scale 3, where they are central. 

In summary, this study proposed a research framework to study the spillover relationships among stocks in the industry chain on different time scales using GARCH-BEKK, heterogeneous network analysis, and wavelet analysis methods. The findings could provide various market participants who are focused on different time scales in one industry with pertinent information. Nonetheless, it should be noted that spillover among stocks is dynamic. Future analyses will be conducted considering this dynamic characteristic in order to explore the changes in the described features over time.

## Figures and Tables

**Table 1:** Different time scales.

Time Scale	Time Horizon (days)	
**D1**	2–4	Short-Term
**D2**	4–8	
**D3**	8–16	Medium-Term
**D4**	16–32	
**D5**	32–64	Long-Term
**D6**	64–128	

**Table 2:** The average influence of stocks with different industry chain attributes.

Chain Attribute	Scale 1	Scale 2	Scale 3	Scale 4	Scale 5	Scale 6
Upstream	12.2	22.6	11.0	10.3	4.9	2.3
Midstream	12.8	21.8	11.6	10.1	6.1	3.1
Downstream	12.1	22.8	11.8	12.2	7.4	3.4
More than one link	13.3	24.0	16.4	12.7	8.3	3.1

**Table 3:** Typical stocks terms of in influence, sensitivity, and intermediary.

Influence						
Scale 1		Scale 2	Scale 3	Scale 4	Scale 5	Scale 6
rank	ID	ID	ID	ID	ID	ID
1	45	45	16	30	16	2
2	43	24	3	17	14	26
3	47	57	43	14	30	20
Sensitivity						
Scale 1		Scale 2	Scale 3	Scale 4	Scale 5	Scale 6
rank	ID	ID	ID	ID	ID	ID
1	45	33	27	32	27	52
2	5	48	53	39	54	53
3	47	49	48	34	53	15
55	9	36	2	7	45	2
56	24	4	14	9	22	42
57	4	30	4	2	21	26
Intermediary						
Scale 1		Scale 2	Scale 3	Scale 4	Scale 5	Scale 6
rank	ID	ID	ID	ID	ID	ID
1	45	23	16	52	7	34
2	20	55	43	30	8	5
3	43	46	48	22	9	17

## Appendix A

**Table A1:** Stocks in the lithium-ion battery industry chain.

ID	Name	Industry Chain Attribute	Stock Code
1	GGEC	downstream	002045.SZ
2	BAOLI NEW	downstream	300116.SZ
3	CAMEL GROUP	downstream	601311.SH
4	DESAYBATTERY	downstream	000049.SZ
5	GREAT POWER	downstream	300438.SZ
6	TOPBAND	downstream	002139.SZ
7	AUCKSUN	downstream	002245.SZ
8	GXHT	downstream	002074.SZ
9	VISION GROUP	downstream	002733.SZ
10	SUNWODA	downstream	300207.SZ
11	JIAWEI ENERGY	downstream	300317.SZ
12	GANFENGLITHIUM	upstream & downstream	002460.SZ
13	CATL	downstream	300750.SZ
14	SMARTER ENERGY	downstream	600869.SH
15	EVE	downstream	300014.SZ
16	DFD	midstream & downstream	002407.SZ
17	NARADA POWER SOURCE	downstream	300068.SZ
18	JSGT	midstream	002091.SZ
19	CAPCHEM	midstream	300037.SZ
20	NBSS	up & mid & downstream	600884.SH
21	TINCI	midstream	002709.SZ
22	YONGTAI TECHNOLOGY	midstream	002326.SZ
23	SHANDONG SHIDA SHENGHUA CHEMICAL GROUP	midstream	603026.SH
24	ZJJH	midstream	600160.SH
25	TONZE	midstream	002759.SZ
26	FULIN PM	midstream	300432.SZ
27	JIANGTE MOTOR	upstream & midstream	002176.SZ
28	XTEMD	midstream	002125.SZ
29	GEM	upstream & midstream	002340.SZ
30	GHKJ	midstream	002741.SZ
31	EASPRING	midstream	300073.SZ
32	FENGYUAN	midstream	002805.SZ
33	DYNANONIC	midstream	300769.SZ
34	RONBAY TECHNOLOGY	midstream	688005.SH
35	BEIT RUI	midstream	835185.BJ
36	NATIONS	midstream	300077.SZ
37	XFH	midstream	300890.SZ
38	KEDA	midstream	600499.SH
39	HNZK ELECTRIC	midstream	300035.SZ
40	PUTAILAI	midstream	603659.SH
41	SINOMATECH	midstream	002080.SZ
42	GREAT SOUTHEAST	midstream	002263.SZ
43	CANGZHOU MINGZHU	midstream	002108.SZ
44	SENIOR	midstream	300568.SZ
45	CHUANGXIN	midstream	002812.SZ
46	ZANGGE MINING	upstream	000408.SZ
47	SINOMINE	upstream	002738.SZ
48	WEIHUA	upstream	002240.SZ
49	YONGXING MATERIALS	upstream	002756.SZ
50	YAHUA GROUP	upstream	002497.SZ
51	ZHEJIANG TIANTIE INDUSTRY	upstream	300587.SZ
52	TLC	upstream	002466.SZ
53	YOUNGY	upstream	002192.SZ
54	TMD	upstream	000762.SZ
55	CTM	upstream	600711.SH
56	HUAYOU COBALT	upstream	603799.SH
57	HANRUI COBALT	upstream	300618.SZ
